# Corrected evaluation of the breech presentation outcome based on etiology of this presentation in congenitally malformed uterus

**DOI:** 10.3389/fmed.2023.1160229

**Published:** 2023-06-21

**Authors:** Slobodan Sekulic, Nebojsa Stilinovic, Branislava Baturan, Anita Krsman, Igor Tesic, Aleksandra Vejnovic, Djordje Petrovic, Zeljka Nikolasevic, Anna Mijavec, Vesna Pesic, Branka Petkovic

**Affiliations:** ^1^Department of Neurology, University Clinical Center of Vojvodina, Novi Sad, Serbia; ^2^Faculty of Medicine, University of Novi Sad, Novi Sad, Serbia; ^3^Department of Pharmacology, Toxicology and Clinical Pharmacology, Faculty of Medicine, University of Novi Sad, Novi Sad, Serbia; ^4^Department of Obstetrics and Gynecology, University Clinical Center of Vojvodina, Novi Sad, Serbia; ^5^Department of Psychology, Faculty of Medicine, University of Novi Sad, Novi Sad, Serbia; ^6^Centrum Medical Center, Ottawa, ON, Canada; ^7^Institute for Biological Research “Sinisa Stankovic“, National Institute of the Republic of Serbia, University of Belgrade, Belgrade, Serbia

**Keywords:** breech presentation, congenitally malformed uterus, outcome, bias, birth

## Abstract

**Background:**

Breech presentation (BP) results from at random filling of the intrauterine cavity, with an equal probability for a BP or cephalic presentation (CP). Each fetus in BP has its “pair” in CP randomly assumed CP. Direct comparison of BP and CP makes bias to less expressed differences between these two groups. It is therefore necessary to subtract the number of fetuses/newborns from the CP set that are identical to the number of fetuses/newborns in the BP set, with identical characteristics, and add this group to the BP set before comparing them to the rest of the CP fetuses/newborns in the matching process.

**Methods:**

The procedure encompasses nine variables in pregnancies with a congenitally malformed uterus (CMU) identified at the Department of Obstetrics (1985–2014): gestational age, birth mass, birth length, head circumference, shoulders circumference, umbilical length, placental weight, newborn mass/newborn length ratio, and newborn mass/placental mass ratio. Firstly, the probability of BP was determined and its relation to gestational age, physical characteristics, and previous presentations. Then direct comparison as well as case–control matching of the CP and BP were performed. Case–control matching was based on either a single specific variable (M1) or all combined variables (M2).

**Findings:**

462 deliveries were identified with CMU. In 81 cases of multiparity, a fetal presentation was found to be an independent event regardless of the previous presentation, gestational age, and newborn physical characteristics. In four types of CMU with 337 deliveries (Bicornuate, Didelphys, Unicornuate, Arcuate), 9 variables with 36 instances of comparison were observed. M1 in 10 instances and M2 in 6 instances showed a statistically significant lower value of breech/random presentation compared with CP. CP have lower value in 2 instances in M1 and 1 in M2. Statistically significant differences were absent without the matching process.

**Interpretations:**

The study confirms the maximum probability for the BP is 50%. The case–control matching procedure shows that it is able to detect the difference between the breech/random presentation and CP, while the classic method of direct comparison was unable to detect any differences. The outcome of the breech/random presentation in CMU should be evaluated with the described case–control matching procedure.

## Introduction

**Figure 1:**
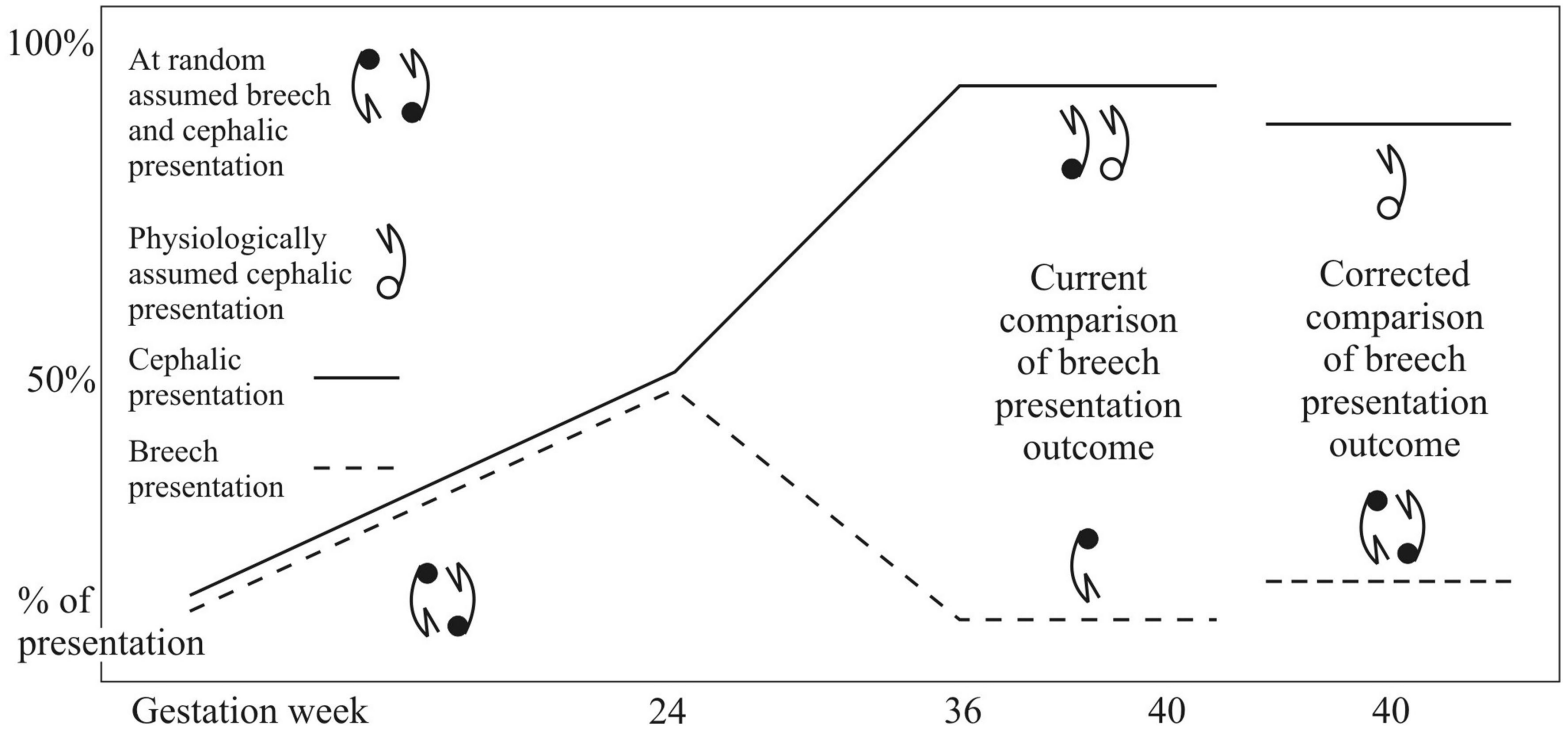
Incidences of fetal presentations during gestation in the general population, etiological mechanisms of the fetal presentations and outcome evaluations of the breech presentation.

In a breech presentation, the fetus lies longitudinally with its buttocks, knees or feet positioned in the isthmic part of the uterus while its head is in the fundal region. Up to the 24th week of gestation, the fetus frequently changes its presentation, and incidences of breech and cephalic presentation are equal. From the 24th to the 36th week of gestation, there is an increase in cephalic presentation with a proportional decrease in breech presentation (1–4). The probability for definitive presentation appears and increases during this period (5–7). After the 36th week of gestation, the incidence of breech (3%) as well as cephalic (96%) presentation remains mainly unchanged (1–6) (Figure 1).

**Figure 2:**
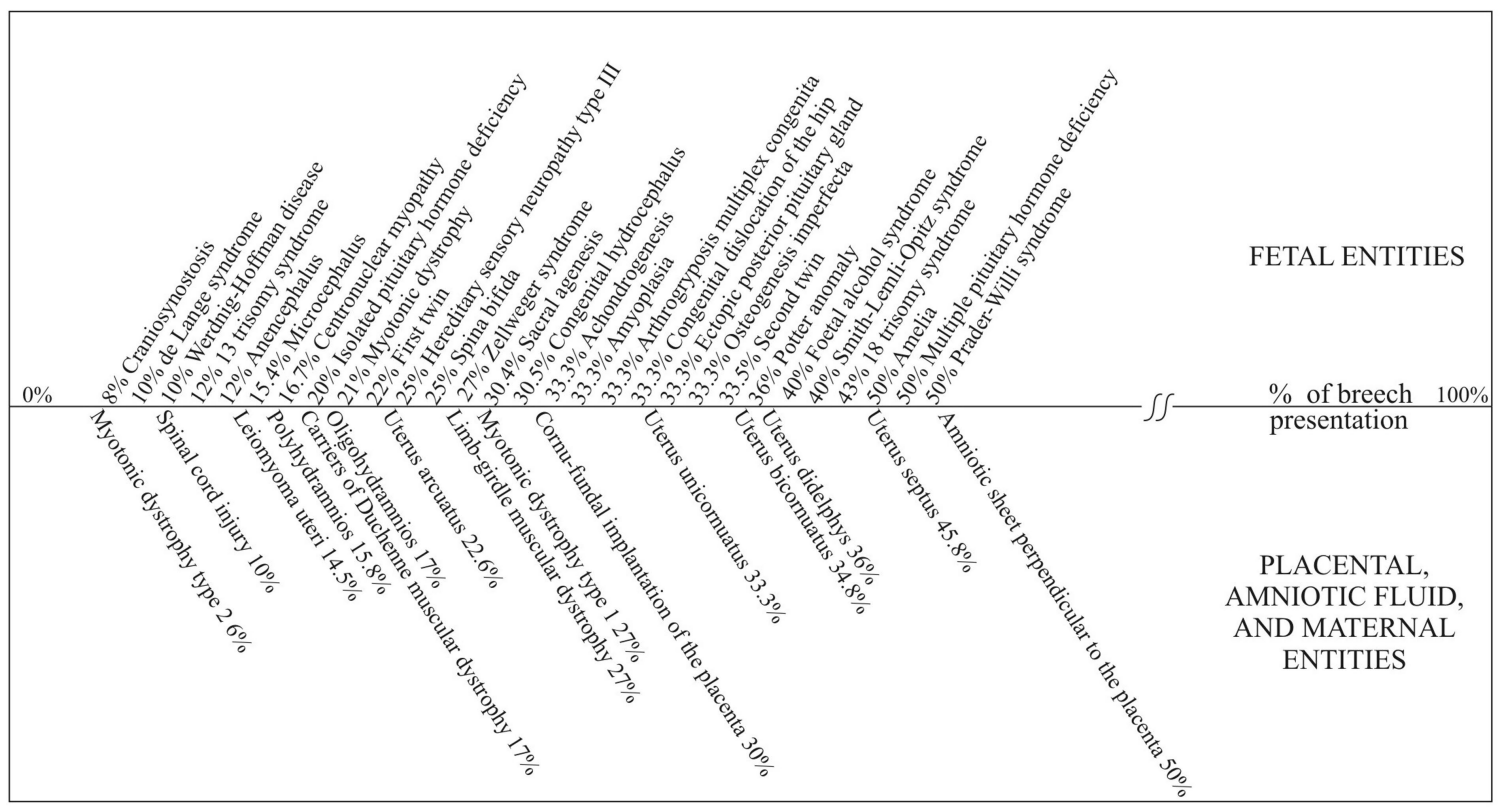
Incidence of breech presentation among various medical entities. Data in figure are from references (7–11).

So far, the outcome of gestations with breech-presenting fetuses has been evaluated based on data obtained by directly comparing the breech and cephalic groups. This current method of comparison was introduced prior to the probability of breech presentation was determined. For example, a study of more than 50 known diseases and medical conditions with the incidence of breech presentation higher than the one which occurs in the general population, revealed that the probability of breech presentation is located within the interval between 3% and 50% (8, 9). The largest part of these medical entities is shown in Figure 2.

The highest possible probability, which is 50% for a breech presentation, shows that it results from a random event, whether the fetus randomly changes between breech and cephalic presentation, or arranges its body parts in the space available. For example, a fetus could assume either a breech or a cephalic presentation in a longitudinally elongated uterus. Therefore, each fetus in a breech presentation has its ‘pair’, which has randomly assumed a cephalic presentation (8, 9). When the incidence of breech presentation is 50%, all fetuses in the breech and cephalic-presenting groups have assumed their presentation randomly. However, when the probability of breech presentation is less than 50%, then the group of cephalic-presenting fetuses is heterogeneous. Some fetuses from this group have assumed a cephalic presentation randomly, while others assume a cephalic presentation as a part of the physiological process, resulting in the majority of the fetuses assuming a cephalic presentation at delivery. The mechanism of this physiological process is still unknown (8, 9).

Compared to cephalic presenting newborns of the same gestational age, breech presenting newborns have: 1. Smaller body lengths (12, 13), 2. Lower body weights (12–14), and 3. Increased proportions of small for gestational age newborns (14, 15). In addition, there are reports of decreased placental weights (12, 13), lower fetal-to-placenta ratio (12), shorter umbilical cords (16), and an increased incidence of congenital malformations (12, 17) in the breech presenting group.

The heterogeneity of the cephalic-presenting group raises the question concerning the validity of the direct comparisons of breech- and cephalic-presenting fetuses/newborns. For example, in the group of fetuses that randomly assume a cephalic presentation, there is an increased incidence of the above-mentioned diseases and medical conditions for the same reason that applies to breech presentation. This makes the difference between the breech-presenting and the cephalic-presenting fetuses/newborns smaller than it should be. The mode of delivery does not influence the physical characteristics of the fetus or the placenta, nor on the inherited and acquired fetal diseases before delivery (8, 9).

Before making a comparison, bias should be eliminated by subtracting from the cephalic presenting group the number of fetuses/newborns with identical characteristics to the number of fetuses/newborns in the breech presentation. This subtracted group of cephalic presenting fetuses/newborns should be added to the breech presenting group before being compared with the rest of the cephalic presenting fetuses/newborns (Figure 1) (8, 9).

To test this new concept, we chose the pregnancies complicated with a congenitally malformed uterus in relation to the characteristics of the newborn and the placenta that are not affected by the delivery. Congenital malformations of the uterus allowed for testing the probability of a breech presentation in repeated pregnancies/deliveries under the same conditions.

The aims of this study were, first, to confirm that breech presentation is a consequence of the random filling of the intrauterine cavity with equal probability for breech and cephalic presentation in CMU. Second, to show that the classical method of direct comparison of the breech and cephalic group has bias towards the less expressed differences between these two groups.

The assumptions are as followed: 1. Maximum probability of breech presentation in CMU is 50%. 2. The new method of comparison for the outcome of breech and cephalic presentations will show a more expressed difference than the classic method for comparison. 3. Using the new method of comparison, there will be differences between the breech and cephalic groups that were not present with the classic method for comparison.

## Materials and methods

### Study population

The research was conducted at the Department of Gynecology and Obstetrics, University Clinical Center of Novi Sad. In accordance with the Helsinki declaration, the study was approved by the Ethical Committee of the Univesity Clinical Center of Vojvodina 00-5/299 – date of issue: May 7, 2015. This was a retrospective cohort study. It included the period from January 1, 1985 to December 31, 2014. The study consisted of all consecutive singleton pregnancies with the following congenital uterine anomalies: septate, unicornuate, bicornuate, didelphys, and arcuate uterus. Congenital uterine anomalies were diagnosed by: hysterosalpingography, ultrasonography, hysterosonography, hysteroscopy, laparoscopy, laparotomy, and magnetic resonance imaging. Each pregnancy was considered an individual case. Because of the study’s retrospective nature, informed consent from patients was unnecessary.

The exclusion criteria was absence of data on the type of the malformed uterus, absence of data on the presentation at delivery, previously performed uteroplastic intervention, presence of myoma uteri, intrauterine adhesions, twin pregnancies, and severe congenital fetal malformations.

The study did not include a control group of infants from noncomplicated cases of cephalic presentation. Regarding the absence of CMU in this group, the matching procedure is not justified.

### Data collection

Data was acquired by reviewing the history of disease and birth protocols. Besides the patient’s full name, they were identified according to their unique personal number. This way, last name changes did not influence the patient’s identification. Data registered was related to the type of the malformed uterus; presentation at birth; gestational age; newborn mass, length, head circumference, shoulder circumference, umbilical length, and placental weight. From the obtained data, two new values were calculated: newborn mass/newborn length ratio and newborn mass/placental mass ratio. Five cohorts were formed. Each cohort represented a particular type of congenitally malformed uterus: uterus didelphis, uterus bicornis, uterus unicornis, uterus arcuatus, and uterus duplex.

### Definitions

Classification of uterine anomalies was made according to the American Fertility Society classification (18). Gestational age at delivery was determined by the last menstrual period and was verified based on the newborn’s neural and physical maturity characteristics.

### Statistical analysis

The IBM SPSS Statistics for Windows, version 23.0, was used for all the analyses (IBM Corp., Armonk, NY, United States). The data set was analysed for normality and outliers using normal plots, with tests and histograms. Samples with values greater than 3 standard deviations than the mean gestational age were removed from further analysis. Cases with extremely low gestational ages were excluded as they affected all other continuous variables of the fetus. They small number produce heterogeneity of the group. After the statistical analysis was done, the two-tailed Fisher’s exact probability or the Pearson Chi-Square test with Yates’s correction for continuity was used to compare the categorical variables between the two groups. McNemar’s test was performed to establish the relationship between the first and the second birth presentation. The Student’s *t*-test or the Mann–Whitney U test was employed to compare continuous variables. The case–control matching procedure was applied to randomly match cases in two different methods. The case–control matching procedure for both means encompassed the following variables: gestational age, birth mass, length, head circumference, shoulders circumference, umbilicus length, placental weight, newborn mass/newborn length ratio, and newborn mass/placental mass ratio. The difference was that in the first procedure, matching cases was based on a single specific variable (M1), and in the second, matching was based on all combined variables (M2). Furthermore, each variable had a tolerance factor (fuzz factor) of 1.5 SD. SPSS options “Give priority to exact matches” and “Maximize execution performance” were applied, while “Randomize case order when drawing matches” was not used. Afterwards, matched cases from the cephalic-presenting group were moved to the breech-presenting group where statistical analysis was completed once more.

## Results

At the Department for Obstetrics and Gynecology, University Clinical Centre of Vojvodina, during the period from the year 1985 until the year 2014, there were 125,240 deliveries. A total of 462 deliveries were identified with women giving birth with congenital uterus malformations, which makes an incidence of 0.37. There were 27 cases that were excluded due to a transverse lie, 11 were excluded due to a myoma uteri, and 5 were excluded due to uteroplastic interventions.

Eighty-one cases of multipara with two deliveries were identified. In cases of multiparty there was no data available for all nine variables except for the fetal presentation data (due to the delivery occuring in another institution and incomplete medical data).

The Chi-Square test 2 × 2 showed no difference in the distribution of cases with breech and cephalic presentation at the first and second delivery in multiparity (Table 1).

**Table 1:** Number of breech and cephalic presentation among different types of congenitally malformed uterus in multiparity at delivery.

Type of malformed uterus	First delivery		Second delivery		χ^2^ test *p* value
	B	C	B	C	
Bicornuate	20	17	19	18	0.815
Didelphys	6	12	4	14	0.456
Septate	5	5	6	4	0.653
Unicornuate	6	2	2	6	0.134
Arcuate	2	6	1	7	1.000
Total	39	42	32	49	0.267

In Table 2, variants of cases for pairs of presentations at the first and the second delivery were presented for different types of congenitally malformed uteri. There was no statistically significant difference in incidence determined for variants of pair presentations at the first and the second birth for any type of CMU. Among 39 cases which had a breech presentation at the first delivery, this presentation was repeated in 16 cases at the mother’s second delivery. From the rest of the 32 cases in breech presentation at second delivery, 16 of them had cephalic presentation at first delivery. The difference in nonrepeating breech presentation at second delivery compared to repeating breech presentation was statistically significant (Chi square test 2 × 2, 39:16 vs. 39:39 *p* value = 0.015 *p* < 0.05).

**Table 2:** Variants of presentation pairs at delivery in multiparity for various types of the congenitally malformed uterus.

Type of malformed uterus	Variants of presentation pairs at delivery in multiparity					χ^2^ test *p* value
	C-C	C-B	B-C	B-B	Total	
Bicornuate	9	8	9	11	37	0.630
Didelphys	9	3	5	1	18	1.000
Septate	1	4	3	2	10	0.519
Unicornuate	2	-	4	2	8	1.000
Arcuate	5	1	2	-	8	1.000
Total	26	16	23	16	81	0.787

C, cephalic presentation; B, breech presentation.

Regarding the physical characteristics of the fetus, placenta and gestational age, there were no significant differences between breech and cephalic group at the first and the second deliveries (Table 3).

**Table 3:** Newborn characteristics in multiparity.

Newborn characteristics	First delivery		*t* test *p* value	Second delivery		*t* test *p* value
	Cephalic presentation	Breech presentation		Cephalic presentation	Breech presentation	
Gestation week	38.52 ± 3.80 WG (34)	39.03 ± 1.80 WG (30)	0.494	38.36 ± 2.69 WG (46)	38.96 ± 2.86 WG (31)	0.361
Birth weight	3038.71 ± 710.53 g (39)	2.973 ± 502.85 g (32)	0.654	3014.02 ± 778.87 g (46)	3139.03 ± 530.79 g (31)	0.404
Birth length	47.28 ± 4.73 cm (25)	47.17 ± 2.24 cm (17)	0.924	48.131 ± 3.39 cm (38)	48.56 ± 2.52 cm (30)	0.546
Head circumference	32.89 ± 3.03 cm (19)	32.75 ± 1.43 cm (16)	0.854	33.72 ± 2.42 cm (37)	33.89 ± 1.63 cm (29)	0.740
Shoulder circumference	35.42 ± 3.64 cm (19)	34.43 ± 2.25 cm (16)	0.336	33.84 ± 4.08 cm (32)	35.86 ± 3.55 cm (25)	0.089

(XX), Number of cases.

Regarding the fetal physical characteristics and gestational age, no statistically significant differences were found between the newborn group that repeated the same presentation from the previous delivery and the group that had a different presentation (Table 4).

**Table 4:** Newborn characteristics at second delivery in case of the same or different presentation on first and second delivery.

Newborn characteristics on the second delivery	The same presentation on first and second delivery (no of cases)	Different presentations on the first and second delivery (no of cases)	*t* test *p* value
Gestation week	38.53 ± 2.83 WG (41)	38.77 ± 2.73 WG (36)	0.705
Birth weight	3081.70 ± 763.83 g (41)	3055.97 ± 617.22 g (36)	0.870
Birth length	48.14 ± 3.20 cm (35)	48.57 ± 2.86 cm (33)	0.558
Head circumference	33.85 ± 2.19 cm (35)	33.83 ± 2.06 cm (29)	0.805
Shoulder circumference	35.21 ± 4.08 cm (32)	34.16 ± 3.85 cm (25)	0.321

(XX), Number of cases.

The data acquired in multiparity indicate that breech presentation is a consequence of the random filling of the intrauterine cavity with an equal probability for a breech or cephalic presentation. The results justify applying a matching procedure to evaluate the breech presentation outcome, which was done in the following part of the work.

Data for the statistical analysis of CMU with all nine variables was available for 350 deliveries. After stratification of the sample and excluding the cases of extreme prematurity, 337 deliveries remained (Table 5). Therefore, in examining the characteristics for each type of congenitally malformed uterus, all included cases had all the mentioned parameters that were being examined.

**Table 5:** Incidence of presentation in various types of the congenitally malformed uterus.

Type of malformed uterus	Breech presentation	Cephalic presentation	Total
	no (%)	no (%)	
Bicornuate	68 (41.21)	97 (58.79)	165
Didelphys	15 (32.60)	31 (67.40)	46
Septate	22 (59.45)	15 (40.55)	37
Unicornuate	14 (40%)	21 (60%)	35
Arcuate	17 (38.63)	37 (61.37)	54
Total	136 (40.36)	201 (59.64)	337

The comparison of before and after matching was not performed in the case of the septate uterus. Taking into consideration the incidence of the breech presentation of ≥50%, sampling bias towards less expressed differences between the breech and the cephalic group is absent according to the concept presented in the introduction. Fetuses/newborns in both groups were taken at random their presentation.

In four types of CMU (arcuate, bicornuate, didelphys, unicornuate), nine variables with 36 instances of comparison were observed before and after the matching (Tables 6–8). No statistically significant difference was present in any of the variables without the matching. When M1 matching was performed, in 10 instances, variables in the breech/random group compared to the cephalic group had statistically significant lower values, while in two instances, the cephalic group had lower values which were statistically significant. In the M matching procedure in 6 instances, variables in the breech/random group compared to the cephalic group had statistically significant lower values. In one instance statistically significant lower values were observed in the cephalic group.

**Table 6:** The matched and unmatched outcome of breech presenting newborns in relation to gestational age, birth weight, and birth length.

	No of cases		Gestation week			Birth weight (gr)			Birth length (cm)		
	B	C	B	C	Sig.	B	C	Sig.	B	C	Sig.
**Arcuate**											
Unmatched	17	37	38.94 ± 2.07	39.83 ± 1.06	0.108	3015.29 ± 580.65	3278.64 ± 543.06	0.125	48.17 ± 2.62	49.56 ± 2.21	0.069
Matched 1	34	20	39.11 ± 1.64	40.30 ± 0.80	**0.001**	3089.70 ± 506.39	3376.00 ± 620.90	**0.090**	48.44 ± 2.46	50.30 ± 1.86	**0.003**
Matched 2	34	20	39.17 ± 1.58	40.20 ± 1.10	**0.008**	3041.17 ± 458.91	3458.50 ± 635.62	**0.015**	48.38 ± 2.13	50.40 ± 2.39	**0.004**
**Bicornuate**											
Unmatched	68	97	38.55 ± 2.20	39.16 ± 1.90	0.068	2930.58 ± 557.60	3079.73 ± 668.27	0.121	48.13 ± 2.59	48.54 ± 3.17	0.359
Matched 1	136	29	38.55 ± 2.17	39.16 ± 1.90	**0.063**	3062.31 ± 616.85	2796.66 ± 638.03	*0.054*	48.00 ± 2.94	50.29 ± 1.99	**0.000**
Matched 2	136	29	38.96 ± 2.03	38.68 ± 2.13	*0.516*	3007.45 ± 613.67	3068.96 ± 698.56	*0.633*	48.48 ± 2.86	47.86 ± 3.29	*0.303*
Didelphys											
Unmatched	15	31	38.40 ± 1.76	38.54 ± 1.82	0.793	2826.66 ± 517.47	2997.09 ± 667.55	0.349	47.26 ± 2.93	47.61 ± 3.75	0.735
Matched 1	30	16	38.76 ± 2.07	38.00 ± 0.89	*0.089*	2853.33 ± 594.87	3106.87 ± 657.18	**0.208**	47.20 ± 3.12	48.06 ± 4.12	**0.471**
Matched 2	30	16	38.33 ± 1.88	38.81 ± 1.60	**0.370**	2825.00 ± 565.89	3610.00 ± 680.86	**0.104**	46.90 ± 3.52	48.62 ± 3.20	**0.102**
Unicornuate											
Unmatched	14	21	38.85 ± 1.35	38.71 ± 2.36	0.822	2771.42 ± 381.39	2920.00 ± 729.23	0.438	46.14 ± 5.14	47.66 ± 2.59	0.319
Matched 1	28	7	38.96 ± 2.16	38.00 ± 0.81	*0.071*	2806.07 ± 553.66	3078.57 ± 818.50	**0.370**	46.67 ± 4.03	48.57 ± 2.50	**0.141**
Matched 2	28	7	38.71 ± 1.99	39.00 ± 2.16	**0.758**	2793.57 ± 612.27	3128.57 ± 571.82	**0.202**	46.71 ± 4.17	48.42 ± 1.27	**0.073**
Septate											
Unmatched	22	15	38.59 ± 2.08	38.60 ± 1.54	0.988	2959.09 ± 513.77	3150.66 ± 519.96	0.277	48.04 ± 2.31	48.93 ± 3.41	0.389

B, breech presentation; C, cephalic presentation; Sig., significance; Bold, increased difference; Bold and Underlined, increased and statistically significant difference; Italic, decreased difference; Italic and Underlined, inverted relation of values.

**Table 7:** The matched and unmatched outcome of breech presenting newborns in relation to head circumference, shoulder circumference and newborn mass/length ratio.

	No of cases		Head circumference (cm)			Shoulder circumference (cm)			Newborn mass/length ratio		
	B	C	B	C	Sig.	B	C	Sig.	B	C	Sig.
**Arcuate**											
Unmatched	17	37	33.76 ± 1.60	34.48 ± 1.40	0.122	35.11 ± 3.03	34.78 ± 2.72	0.702	62.14 ± 9.18	65.87 ± 8.21	0.163
Matched 1	34	20	33.55 ± 1.28	35.45 ± 0.99	**0.000**	34.41 ± 2.57	35.70 ± 3.06	*0.123*	63.33 ± 7.95	67.02 ± 2.10	**0.150**
Matched 2	34	20	33.79 ± 1.40	35.05 ± 1.31	**0.002**	35.05 ± 2.81	34.60 ± 2.83	**0.568**	62.61 ± 7.49	68.24 ± 9.42	**0.029**
**Bicornuate**											
Unmatched	68	97	33.67 ± 2.54	33.77 ± 1.88	0.790	34.33 ± 2.95	34.70 ± 3.49	0.473	60.49 ± 8.91	68.56 ± 58.08	0.167
Matched 1	136	29	33.64 ± 2.00	34.21 ± 2.82	**0.312**	34.04 ± 3.12	37.37 ± 2.60	**0.000**	60.64 ± 8.92	62.92 ± 11.23	**0.145**
Matched 2	136	29	33.69 ± 2.20	33.89 ± 2.02	**0.640**	34.45 ± 3.10	35.00 ± 4.01	*0.497*	65.55 ± 47.54	63.76 ± 12.82	*0.706*
**Didelphys**											
Unmatched	15	31	33.33 ± 1.49	33.32 ± 2.24	0.985	33.20 ± 3.14	34.67 ± 3.23	0.150	59.43 ± 7.74	62.32 ± 10.16	0.293
Matched 1	30	16	33.16 ± 1.51	33.62 ± 2.75	*0.544*	33.06 ± 3.12	36.31 ± 2.33	**0.000**	63.78 ± 8.26	56.88 ± 10.15	***0.028***
Matched 2	30	16	32.93 ± 1.81	34.06 ± 2.20	*0.091*	33.93 ± 2.93	34.68 ± 3.82	*0.498*	59.77 ± 8.57	64.40 ± 10.54	**0.143**
**Unicornuate**											
Unmatched	14	21	32.64 ± 1.33	33.38 ± 2.10	0.214	34.28 ± 2.52	34.14 ± 3.77	0.894	61.03 ± 13.38	60.72 ± 12.62	0.947
Matched 1	28	7	32.64 ± 1.31	34.85 ± 2.67	**0.072**	33.57 ± 3.12	36.71 ± 2.87	***0.029***	61.29 ± 12.12	59.07 ± 15.90	**0.739**
Matched 2	28	7	32.92 ± 1.92	33.71 ± 1.49	*0.266*	34.07 ± 3.31	34.71 ± 3.40	*0.664*	60.89 ± 12.53	60.69 ± 14.30	*0.972*
**Septate**											
Unmatched	22	15	33.22 ± 1.54	34.13 ± 1.99	0.128	34.68 ± 3.21	35.66 ± 3.19	0.365	61.30 ± 8.49	64.13 ± 7.63	0.301

B, breech presentation; C, cephalic presentation; Sig., significance; Bold, increased difference; Bold and Underlined, increased and statistically significant difference; Italic, decreased difference; Italic and Underlined, inverted relation of values; Italic, Bold, and Underlined, inverted relation of values with a statistically significant difference.

**Table 8:** The matched and unmatched outcome of breech presenting group in relation to umbilical length, placental weight, newborn mass/placental mass ratio.

	No of cases		Umbilical length (cm)			Placental weight (gr)			Newborn mass/placental mass ratio		
	B	C	B	C	Sig.	B	C	Sig.	B	C	Sig.
**Arcuate**											
Unmatched	17	37	60.64 ± 5.18	63.48 ± 7.24	0.108	524.11 ± 69.28	559.45 ± 72.68	0.096	5.72 ± 0.67	5.88 ± 0.75	0.441
Matched 1	34	20	60.44 ± 4.97	66.25 ± 7.85	**0.006**	527.35 ± 67.70	584.00 ± 68.77	**0.005**	5.71 ± 0.71	6.04 ± 0.72	**0.106**
Matched 2	34	20	61.17 ± 5.45	65.00 ± 8.09	**0.071**	535.29 ± 71.53	570.50 ± 71.48	**0.088**	5.70 ± 0.68	6.06 ± 0.76	**0.091**
**Bicornuate**											
Unmatched	68	97	60.42 ± 6.64	60.79 ± 7.54	0.742	522.35 ± 85.83	544.12 ± 95.01	0.127	5.62 ± 0.68	6.18 ± 5.17	0.288
Matched 1	136	29	60.47 ± 6.58	61.86 ± 9.72	**0.485**	529.07 ± 81.86	569.25 ± 126.39	**0.122**	5.61 ± 0.76	5.80 ± 1.22	**0.258**
Matched 2	136	29	60.73 ± 6.98	60.20 ± 8.10	*0.746*	532.27 ± 86.22	548.62 ± 114.82	*0.474*	6.01 ± 4.36	5.67 ± 1.30	*0.445*
**Didelphys**											
Unmatched	15	31	62.06 ± 5.33	62.41 ± 7.69	0.858	518.66 ± 90.06	535.48 ± 73.83	0.536	5.50 ± 0.88	5.60 ± 1.04	0.741
Matched 1	30	16	61.16 ± 5.20	64.43 ± 9.24	**0.205**	540.33 ± 81.72	510.62 ± 71.69	*0.211*	5.43 ± 0.92	5.82 ± 1.09	**0.240**
Matched 2	30	16	61.43 ± 5.90	63.93 ± 8.56	**0.307**	521.00 ± 82.85	546.87 ± 70.21	**0.272**	5.43 ± 0.82	5.82 ± 1.22	**0.268**
**Unicornuate**											
Unmatched	14	21	60.07 ± 5.31	63.71 ± 6.30	0.075	517.14 ± 53.55	526.66 ± 96.40	0.711	5.36 ± 0.54	5.51 ± 0.78	0.497
Matched 1	28	7	61.21 ± 5.24	66.42 ± 7.95	**0.048**	541.78 ± 75.67	447.14 ± 56.48	***0.003***	5.38 ± 0.73	5.75 ± 0.39	**0.085**
Matched 2	28	7	61.07 ± 5.82	67.00 ± 5.19	**0.025**	524.28 ± 82.75	517.14 ± 80.35	*0.839*	5.37 ± 0.53	5.72 ± 1.07	**0.393**
**Septate**											
Unmatched	22	15	59.68 ± 6.62	58.26 ± 7.36	0.774	525.45 ± 76.51	579.33 ± 59.45	0.028	5.65 ± 0.81	5.44 ± 0.69	0.418

B, breech presentation; C, cephalic presentation; Sig., significance; Bold, increased difference; Bold and Underlined, increased and statistically significant difference; Italic, decreased difference; Italic and Underlined, inverted relation of values; Italic, Bold, and Underlined, inverted relation of values with a statistically significant difference.

Regarding the types of CMU, the arcuate uterus showed that the characteristics of the fetuses were significantly worse after the comparison. There was a statistically significant worsening of characteristics with regards to the physical characteristics of the fetus and placenta that were also present in the other three types of congenital malformations, but to a lesser degree.

## Discussion

The incidence of CMU in this study, 0.37% is on the lower end of the scale when compared to other studies: 0.5%, 3.2%, and 5.4% (19–21). The incidence of a breech presentation among various types of the congenitally malformed uterus presented in this study (Table 5) were in accordance with the data presented in other studies. Studies with more than 20 cases for a particular type of malformed uterus showed that incidences of breech presentation were: 1. Uterus bicornuate 34.78%, 35%, 41.7%, and 46.47% (8, 22–24); 2. Uterus didelphys 36.4% and 41.17% (8, 23); 3. Uterus unicornuate 34% and 55.6% (23, 24); 4. Uterus arcuate 13, 22.58, and 40% (8, 22, 24); and 5. Uterus septate is an exception where the value in this study of 59.45% was higher than in other studies 35%, 38.33%, 45.71%, and 47.4% (8, 22–24).

In multiparity with CMU, the difference between the incidence of breech and cephalic presentations between the first and the second delivery were not statistically significant. This result was achieved despite the fact that subsequent deliveries did not repeat the same fetal presentation as on the first delivery. Pairs of presentations in multiparity were the result of independent events, during which the first and second birth had the same probability for a breech or a cephalic presentation independent of fetal physical characteristics and gestational age.

The results of this study confirm the findings of earlier studies, that the maximum probability for a breech presentation is 50% (8, 9).

Binomial distribution allows for values over 50% in individual series when the probability for the breech is close to or exactly 50%, similar to the probability of tossing a coin. For example, when considering a series with fewer tries in tossing the coin, the probability of a tail can be over or under 50%. When there is an infinite number of coin tosses, the incidence will be reaching a value that is closest to the ideal distribution 50:50 (25). We have identified only one study with a significant number of cases in which there was an incidence of breech presentation that was significantly higher than 50% (26). The authors of that study stated that not all the cases had had a routine examination in regard to CMU. However, detailed diagnostic searches were performed only when there were complications at birth, which included breech presentation. This resulted in bias regarding a higher frequency of breech presentation. In accordance with this, there was a low incidence of CMU, 0.13% in that specific study (26).

The fetus changes its presentation/lie from breech to cephalic and vice versa using a variety of movements, including kicking, locomotion, body twisting, and trunk and lower extremity stretching (27). The cephalic version of breech fetuses in cases of the congenitally malformed uterus is not recommended. A published study shows that the external version of breech to cephalic presentation was successful in one of 12 attempts (21). Furthermore, congenital malformations of the uterus present a contraindication for the fetus’s external or internal version because of insufficient intrauterine space volume (28). A likely explanation for the etiology of breech presentation in the case of CMU is that in the period up to 24 weeks of gestation, when the fetuses have randomly assumed a presentation (Figure 1), they have outgrown the malformed uteruses. The fetuses remained compressed in the uterine cavity before or at the beginning of the gestational period, characterized by the exclusive increase in the incidence of cephalic presentation from 24–36 WG (Figure 1). This could explain why the fetuses assume a breech presentation at random and why the probability of breech presentation in CMU is no higher than 50%. Thus, it would be interesting to further explore the incidence of presentation during gestation for each type of CMU.

To our knowledge, there were no studies available that examined the differences between breech and cephalic presenting newborns in CMU (22–24, 26). As a rule, the outcomes of CMU were examined regardless of the presentation.

The case–control matching procedure randomly matched cases and controls based on specific criteria. In other words, matching is used to balance the distributions of observed (and possibly confounding) covariates. Ideally, one would match each experimental subject with a control subject that was an exact match on each of the observed covariates. As the number of covariates increases or the ratio of the number of control subjects to examined subjects decreases, which was the case in our study, it becomes less likely that an exact match would be found for each examined subject. When the matching procedure was based on a single variable, it was easier to find a corresponding case. This was reflected in a larger number of statistically significant differences in M1 matching than in M2.

The case–control matching procedure showed that it is able to detect the difference between the breech/random and the cephalic group of newborns, while the classic method of direct comparison was unable to detect any differences. Some authors consider the arcuate uterus a minimal deviation in the anatomy of the uterus and do not consider it in the outcome of the pregnancy along with the other types of CMU (23, 29). The presented data from the matching procedure for the arcuate uteri, clearly showed that the physical characteristics of the newborn in a breech presentation, were significantly worse than those in the cephalic presentation. It should be noted that in arcuate uteri metroplasty leads to improvement of uterine artery Doppler velocimetry indices, i.e., better uterine perfusion (30). Our data suggest that it is necessary to reconsider the clinical approach in the prognosis, outcome, and management of breech/random presentation in case of CMU. Newborns from breech/random group compared to cephalic group, showed poorer values of body development parameters. In CMU it is necessary to investigate trophic factors such as diminished uterine blood flow, insufficient placental function, in relation to fetal presentation using matching process.

CMU are associated with increased incidence of: missed abortion, prematurity, intrauterine growth retardation, still birth, preterm premature rupture of membranes, postural deformation of the fetus (23, 31). Incomplete data in this study prevented investigation of mentioned entities regarding fetal presentation.

## Conclusion

The study confirms that breech presentation in CMU is a consequence of the random filling of the intrauterine cavity with an equal probability for a breech or cephalic presentation. The case–control matching procedure shows that it is able to detect the difference between the breech/random presentation and CP, while the classic method of direct comparison was unable to detect any differences. The outcome of the breech/random presentation in CMU should be evaluated with the described case–control matching procedure. Applying the matching procedure to evaluate breech presentation outcome in any medical entity with increased incidence of breech presentation than in general population, would shed new light on the deviation from physiological norm and validity of current management of breech presentation.

## Data availability statement

The raw data supporting the conclusions of this article will be made available by the authors, without undue reservation.

## Ethics statement

The studies involving human participants were reviewed and approved by University Clinical Center of Vojvodina. Written informed consent was not provided because retrospective study.

## Author contributions

SS, NS, ZN, BP, and VP: concept and design of the study. SS, BB, AK, IT, DP, and AV: gathering data from the hospital archive, search of literature. NS and ZN: statistical analysis. SS, BB, AK, IT, AV, BP, VP, and AM: interpretation of relevant literature. SS and AM: translation to English language. All authors contributed to the article and approved the submitted version.

## Funding

This work was supported by Ministry of Science, Technological Development and Innovation of the Republic of Serbia, grant numbers 451-03-47/2003-01 and 451-03-47/2023-01/200007.

## Conflict of interest

The authors declare that the research was conducted in the absence of any commercial or financial relationships that could be construed as a potential conflict of interest.

## Publisher’s note

All claims expressed in this article are solely those of the authors and do not necessarily represent those of their affiliated organizations, or those of the publisher, the editors and the reviewers. Any product that may be evaluated in this article, or claim that may be made by its manufacturer, is not guaranteed or endorsed by the publisher.
